# College Students with Oral Habits Exhibit Worse Psychological Status and Temporomandibular-Related Quality of Life: A Correlational Study

**DOI:** 10.1155/2022/6079241

**Published:** 2022-05-11

**Authors:** Wenke Yang, Xin Xiong, Yange Wu, Yufan Zhu, Jiaqi Liu, Chengxinyue Ye, Qinlanhui Zhang, Jun Wang

**Affiliations:** National Clinical Research Center for Oral Diseases, State Key Laboratory of Oral Diseases, Department of Orthodontics, West China Hospital of Stomatology, Sichuan University, No. 14, Section 3, Ren Min South Road, Chengdu 610041, China

## Abstract

**Purpose:**

To evaluate the relationship between oral habits, psychological status, and temporomandibular-related quality of life among college students.

**Materials and Methods:**

An online questionnaire was sent to college students who were willing to participate in this anonymous survey, which contained questions about the demographic characteristics of the participants, the Patient Health Questionnaire for Depression and Anxiety (PHQ-4), the Fonseca Anamnestic Index (FAI), and the Oral Health Impact Profile for temporomandibular disorders (OHIP-TMDs).

**Results:**

A total of 505 valid questionnaires were collected from 200 males and 305 females (a mean age of 21.81 ± 2.81 years). The prevalence of oral habits in college students was 58% (294/505). Female gender (odds ratio (OR) 1.786) and having oral habits (OR 1.893) were associated with depression and anxiety. Medical students had significantly less depression and anxiety (OR 0.459) than nonmedical students. The possibility of suffering from temporomandibular disorder (TMDs) as evidenced by the OHIP-TMDs score was associated with female gender (OR 1.989) and having oral habits (OR 3.482). Students with oral habits had higher OHIP-TMDs scores.

**Conclusion:**

More than half of the college students surveyed had specific oral habits, with a higher prevalence in women than in men. Having oral habits was related to a worse psychological status, higher risk of TMD, and worse temporomandibular-related quality of life.

## 1. Introduction

Temporomandibular disorders (TMDs) represent a group of diseases characterized by pain and dysfunction of the masticatory muscles and the temporomandibular joint (TMJ) [1, 2]. TMDs can affect patients' quality of life considerably [3], and the prevalence of TMDs was found to vary from 7.3% to 33% in previous studies [4–6]. The common symptoms of TMDs include jaw pain or dysfunction, earache, headache, and facial pain [7]. However, the etiology of TMD is numerous and complex and may result from the interplay of age, sex, occlusion, parafunctional habits, and psychological problems [2, 8]. Several biological and psychological risk factors have been identified to be associated with TMDs [1]. It has also been proposed that anxiety and depression could contribute to the development of TMDs [9].

At present, the majority of studies on TMDs and their risk factors have focused on children and adolescents or older adults [4, 6, 10, 11]. There have been few studies specifically focused on college students. The college period is an important transitional stage from school to society, and many college students experience much mental pressure [12]. Previous studies showed that TMDs are more likely to occur in college students and are significantly associated with anxiety and depression [13]. More attention should be paid to college students concerning their psychological status and temporomandibular-related quality of life.

This study specifically focused on college students and aimed to elucidate the correlation between oral habits, psychological status, and TMD-related quality of life. The major area of study of the students was also considered in this survey to explore its relationship with psychological stress and TMD-related quality of life. Unlike the previous studies on this topic, online questionnaires were used to collect and analyze data in our study. This methodology not only resulted in easy-to-access information being available and a high participation rate from college students because of their tendency to spend time online but is also convenient for researchers to process the data with high efficiency. We hypothesized that students with oral habits would be prone to worse psychological status, signs and symptoms of TMD, and worse TMD-related quality of life.

## 2. Materials and Methods

### 2.1. Participants

This study met the requirements of the Declaration of Helsinki. The study protocol was approved by the Institutional Review Board of the West China Hospital of Stomatology (Approval no. WCHSIRB-2020-419). Participants were recruited from the university's undergraduate to graduate populations at several different places within the campus: for example, the entrance to the library, the campus gate, and the roads. The students were identified as such by presenting their student ID cards. The students who were willing to participate in the anonymous survey filled in the questionnaires. Participants majored in both medical and nonmedical fields. We counted medical specialties including stomatology, clinical medicine, nursing, pharmacy, and medical technology. The participation in this study was completely voluntary, and informed consent was obtained from all subjects.

Inclusion criteria were college students who aged from 17 to 35 years, including undergraduates, master's degree students, and doctoral students. Exclusion criteria were as follows: (1) incomplete or obviously unreasonable questionnaire responses; (2) filling in the questionnaire for more than 15 minutes; (3) history of TMJ treatment; (4) history of orofacial trauma; (5) systemic diseases; (6) diagnosed with mental illness. According to a reported 54% prevalence of symptoms of TMDs [2], based on a 95% confidence level with a 5% margin of error for the confidence interval, the minimum sample size of 382 subjects was determined.

### 2.2. Data Collection

**Figure 1:**
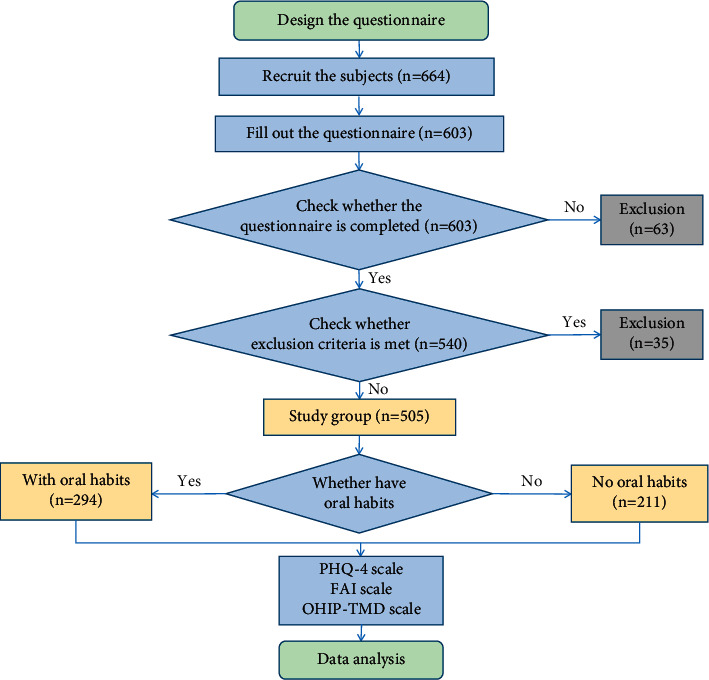
The flowchart of the study.

The questionnaire consisted of four parts and was closed-ended. All questions had two to six answers from which the participants could choose. The questionnaire was uploaded to “Questionnaire Star,” the most widely used questionnaire website in China. The subjects completed the online anonymous questionnaire by clicking the link we sent after being explained in person about the purpose and content of the study and giving verbal informed consent. The questionnaire was self-administered and was completed anonymously, thus ensuring the confidentiality of the subjects. A flow chart depicting the study is shown in Figure 1.

The first part of the questionnaire collected the demographic characteristics of the participants, including gender, age, education level (undergraduate, master's, or doctoral degree), and major (medical student or not), and also contained questions about oral habits. The oral habits investigated mainly included clenching teeth, sleep bruxism, unilateral chewing, biting hard food, and opening the mouth too widely while yawning.

The second part of the questionnaire was the Patient Health Questionnaire for Depression and Anxiety (PHQ-4) [14]. The reliability and validity of PHQ-4 have been demonstrated to be suitable for screening for depression and anxiety in the general population [15]. Responses to the four stem questions were scored as 0 points (“not at all”), 1 point (“several days”), 2 points (“more than half the days”), or 3 points (“nearly every day”). Therefore, the total score on this composite measure ranged from 0 to 12 points. With a total score of more than 2 points, a patient was considered as having psychological distress [16].

The third part of the questionnaire was the Fonseca Anamnestic Index (FAI), which consisted of 10 questions pertaining to jaw-movement difficulties, orofacial pain, TMJ sounds, parafunctional habits, malocclusion perception, and emotional stress [17]. The Chinese version of the FAI has been demonstrated to have acceptable reliability and good validity [18]. This index was created with 10 items with 3 answers each, scored as follows: 10 points (“yes”), 5 points (“sometimes”), and 0 points ('‘no”) [19]. The final score of the instrument was determined by the sum of the scores of all items, allowing the following classifications: absence of signs and symptoms of TMDs (0–15 points), mild TMDs (20–45 points), moderate TMDs (50–65 points), and severe TMDs (70–100 points) [19]. The diagnosis of TMDs was based on the FAI. A score of 0–15 points was considered to be free of TMDs, while a score above 20 points was considered to have TMDs.

The last part of the questionnaire was the Oral Health Impact Profile for TMDs (OHIP-TMDs), with 22 questions. Each question in the OHIP-TMDs section was scored on a five-point ordinal response scale of 0 points (“never”), 1 point (“hardly ever”), 2 points (“often”), 3 points (“fairly often”), and 4 points (“very often”) [20]. The Chinese version has shown good validity and reliability, thus providing a valuable instrument [21]. The total OHIP-TMDs scores are the sum of the 22 questions. Higher total OHIP-TMDs scores indicate worse temporomandibular-related quality of life [22].

### 2.3. Statistical Analysis

Descriptive statistics were performed to characterize the college subjects. Measurement data are represented by mean ± standard deviation (SD), while count data are represented by the frequency and composition ratio. The presence of oral habits was considered as an independent variable. The presence of psychological distress and the diagnosis of TMD, which were binary variables, were considered as dependent variables in bivariate logistic regression analysis to assess their association with the presence of oral habits. The odds ratio (OR), 95% confidence intervals (CIs) for OR, and *P* value were calculated by bivariate logistic regression. The score on the OHIP-TMDs, taken as a continuous dependent variable, was analyzed by multivariate linear regression. The coefficients, 95% confidence intervals (CI) for beta, and *P* values were calculated by multivariate linear regression. The demographic variables were considered as the possible confounding factors. Statistical analyses were performed using SPSS (IBM SPSS Statistics version 21.0, IBM Corp., Armonk, New York, USA) with a two-tailed *α* significance level of 0.05.

## 3. Results

A total of 664 questionnaires were sent to university students, of which 603 questionnaires were completed. After discarding those with incomplete or obviously illogical responses, 505 questionnaires were included in the study, with a response rate of 90.81% and an effective recovery rate of 83.75%. According to the presence or absence of oral habits, the participants were divided into two groups. There were 294 subjects in the group with oral habits and 211 subjects in the group without oral habits. The prevalence of oral habits in females (65.25%) was higher than that in males (47.50%). There were no statistical differences in other demographic variables between the two groups (Table 1).

On the PHQ-4, the FAI, and the OHIP-TMDs scales, the average score of the subjects with oral habits was statistically higher than that of those without (adjusted *P* < 0.001) (Table 2). The evaluation of psychological distress showed that 65.36% of the subjects with oral habits suffered from depression and anxiety. A greater proportion of students with oral habits suffered from TMDs than students without oral habits (*P* < 0.001).

As shown in Table 3, the unadjusted association for depression and anxiety showed a possible positive association (*P* < 0.10) with female gender (OR 1.763; 95% CI 1.229–2.528) and having oral habits (OR 1.938; 95% CI 1.353–2.774) and a possible negative association with medical-related majors (OR 0.553; 95% CI 0.373–0.762). According to the multivariate regression model, having oral habits (OR 1.893; 95% CI 1.304–2.748) and female gender (OR 1.786; 95% CI 1.224–2.607) still showed significant associations with the psychological state. The results showed that college students having oral habits were nearly twice as likely to have anxiety or depression as those without oral habits.

In Table 4, the unadjusted association for the presence of TMDs showed a possible association (*P* < 0.10) with gender (OR 2.322; CI 1.601–3.367), master's degree student status (OR 1.425; 95% CI 0.951–2.137), and oral habits (OR 3.795; 95% CI 2.586–5.567). In the multivariate regression analysis, female gender (OR 1.989; 95% CI 1.349–2.935) and having oral habits (OR 3.482; 95% CI 2.359–5.138) remained significant.

The correlation analysis between the independent variables and the summed scores of OHIP-TMDs showed that only gender (*P*=0.021) and having oral habits (*P* < 0.001) correlated with the dependent variable (Table 5). In the multivariate linear regression, only oral habits had a significant association with the OHIP-TMDs score (adjusted *P* < 0.001).

## 4. Discussion

This study focused on the relationship between oral habits, psychological distress, TMDs, and TMJ-related quality of life among college students. In the group that reported oral habits, women made up more than two-thirds (67.69%) of the participants. There was a considerably larger percentage of women (65.25%) with oral habits than men (47.50%). The reason might be that women are more likely to develop psychological distress than men, which is one of the risk factors for TMDs [23].

Over three-fifths of the surveyed students majoring in medical fields (62.12%) reported oral habits. However, the prevalence of oral habits in subjects majoring in nonmedical fields was almost half. The reason for the higher prevalence among medical students might be that medical students have more knowledge about oral health than nonmedical students [24], and they might focus more on their own health problems [25], so they might be more aware of their oral habits.

The psychological status of subjects with oral habits was on average worse than that of subjects without oral habits. It could be inferred that having oral habits is a symptom of depression and anxiety, or conversely that depression and anxiety contribute to developing oral habits [26]. Further research is needed to confirm the nature of the relationship between psychological state and oral habits. In multivariate regression analysis, gender and major were indicated to be related to the psychological status as well. Females were more prone to develop mental distress, which was consistent with previous studies [11]. Another interesting discovery was that the students majoring in nonmedical fields were more likely to have depression and anxiety than medical students (Table S1). This finding differs from previous studies [27]. One possible reason could be that the sample group was only selected from one university, and there may be some differences between the sample population structure and the overall population structure. Another possible reason is that medical students have knowledge about psychology and may have developed techniques to improve their psychological status.

The presence of oral habits was associated with a higher prevalence of TMDs, consistent with a previous study [28]. For instance, tooth clenching is a risk factor for myofascial temporomandibular disorders (M-TMDs) that increases jaw muscle pain levels in M-TMD patients, and it is associated with significantly higher 5-HT levels and lower blood flow in the masseter muscles [29]. This pain causes discomfort in patients and worsens TMJ-related quality of life. In addition, female gender had a stronger association with TMDs than male gender, which is in accordance with a previous study that found that women had almost two times greater risk of developing TMDs than men [11]. Interestingly, although depression and anxiety were more common among nonmedical students than medical students, there was no statistical difference in the prevalence of TMDs between the two groups (Table S1). One reason may be that the mental status is only one of several factors contributing to the development of TMDs, not a decisive one. Few studies have been conducted on the prevalence of TMDs in these two groups of people, which may be considered for further study.

The subjects with oral habits reported worse temporomandibular-related quality of life. People with oral habits might be prone to damaging the TMJ and the masticatory muscles, causing discomfort in the TMJ area and thus impairing the quality of life. Apart from oral habits and gender, other factors had little correlation with the total scores of OHIP-TMDs and could not be analyzed by multivariate linear regression. According to the linear regression model of the OHIP-TMDs scores, only the presence of oral habits appeared to be related to worse temporomandibular-related quality of life. As shown in previous studies, diurnal clenching was strongly associated with TMD symptoms and also affected the quality of life [28]. The present model, considering the oral habits and other demographic factors, could not predict the total scores of temporomandibular-related quality of life well, indicating there may be other relevant factors that we did not include in this study. Further studies could take other possibly associated factors such as emotional status and sleep quality into account [30].

According to the biopsychosocial model, TMDs are not only the result of the interaction of biological and psychological factors but also the result of social factors. This study will be of great help in exploring the causes of TMDs related to psychological status. It also helps in understanding the prevalence of TMDs and provides new ideas for the prevention and treatment of TMDs among college students. Therefore, when college students seek medical treatment for TMD symptoms, doctors can perform more personalized counseling by taking their identities as college students into account. Besides, these results can give us a hint that university authorities could take some measures to improve the psychological health of college students, such as offering a relevant medical curriculum.

This study also had some limitations. Since it was a cross-sectional questionnaire study, it was easy to implement and was easy to replicate, but no cause-and-effect relationship could be inferred from the results. Further longitudinal studies with oral examinations should be conducted to clarify which factor(s) may precipitate others. Besides, the volunteers were recruited only from one university in one city in China, which could result in overestimating or underestimating the scores of university students more broadly and limiting the generalizability of our results. Further research could be conducted to expand the sample population to different regions, universities, and ethnic groups.

## 5. Conclusions

Over half of the college students surveyed had specific oral habits, and women had a higher prevalence of oral habits than men in the present study. Having oral habits was associated with a worse psychological status, higher risk of TMD, and worse temporomandibular-related quality of life.

## Figures and Tables

**Table 1:** Participant demographics.

Variable	With oral habits (*N* = 294)	No oral habits (*N* = 211)	Total	*P*
*Age (mean* *±* *SD)*	21.79 ± 2.61	21.84 ± 3.06	21.81 ± 2.81	0.834
*Gender (N (%))*				<0.001^*∗∗∗*^
Male	95 (47.50%)	105 (52.50%)	200 (100%)	
Female	199 (65.25%)	106 (34.75%)	305 (100%)	
*Major (N (%))*				0.063
Medical	164 (62.12%)	100 (37.88%)	264 (100%)	
Nonmedical	130 (53.94%)	111 (46.06%)	241(100%)	
*Education level (N (%))*				0.442
Undergraduate	173 (56.72%)	132 (43.28%)	305 (100%)	
Master's	86 (62.77%)	51 (37.23%)	137 (100%)	
Doctoral	35 (55.56%)	28 (44.44%)	63 (100%)	

The total population (*N* = 505), subset of participants with oral habits (*N* = 294), and subset of participants without oral habits (*N* = 211) are given. ^*∗∗∗*^*P* < 0.001.

**Table 2:** Average PHQ-4, FAI, and OHIP-TMDs scores and diagnosis of psychological distress and TMD in participants based on the PHQ-4 and FAI (*N* = 505).

Variable	With oral habits (*N* = 294)	No oral habits (*N* = 211)	Total	*P*
Average score (points)				
The PHQ-4	3.38 ± 2.60	2.57 ± 2.64	3.04 ± 2.64	<0.001^*∗∗∗*^^,a^
The FAI	23.83 ± 18.09	12.63 ± 15.37	19.95 ± 17.87	<0.001^*∗∗∗*^^,a^
The OHIP-TMDs	9.66 ± 12.88	5.08 ± 10.92	7.74 ± 12.30	<0.001^*∗∗∗*^^,a^
Psychological distress				<0.001^*∗∗∗*^
Without	111 (49.33%)	114 (50.67%)	225 (100%)	
With	183 (65.36%)	97 (34.64%)	280 (100%)	
TMD				<0.001^*∗∗∗*^
No	124 (44.44%)	155 (55.56%)	279 (100%)	
Mild	136 (74.73%)	46 (25.27%)	182 (100%)	
Moderate	26 (76.47%)	8 (23.53%)	34 (100%)	
Severe	8 (80.00%)	2 (20.00%)	10 (100%)	

^*∗∗∗*^ *P* < 0.001. ^a^Adjusted *P* value using Bonferroni correction.

**Table 3:** Univariate and multivariate logistic regression analysis of variables associated with the PHQ-4 (*N* = 505).

Independent variables	Univariate regression		Multivariate regression	
	*P* value	Odds ratio (95% CI)	Adjusted *P* value	Odds ratio (95% CI)
Age	0.103	0.949 (0.891–1.011)		
Gender				
Male		Reference		Reference
Female	0.002^*∗∗*^	1.763 (1.229–2.528)	0.003^*∗∗*^	1.786 (1.224–2.607)
Major				
Nonmedical		Reference		Reference
Medical	<0.001^*∗∗∗*^	0.533 (0.373–0.762)	<0.001^*∗∗∗*^	0.459 (0.316–0.666)
Education level				
Undergraduate	0.358	Reference		
Master	0.152	0.744 (0.496–1.115)		
Doctor	0.754	0.916 (0.531–1.582)		
Oral habits				
Without		Reference		Reference
With	<0.001^*∗∗∗*^	1.938 (1.353–2.774)	<0.001^*∗∗∗*^	1.893 (1.304–2.748)

A total score of 0–2 was considered normal, whereas a score of 3–12 was taken as indicative of mental distress. ^*∗∗*^*P* < 0.01, and ^*∗∗∗*^*P* < 0.001.

**Table 4:** Univariate and multivariate logistic regression analysis of variables associated with the presence of TMDs (*N* = 505).

Independent variables	Univariate regression		Multivariate regression	
	*P* value	Odds ratio (95% CI)	Adjusted *P* value	Odds ratio (95% CI)
Age	0.838	1.007 (0.946–1.072)		
Gender				
Male		Reference		Reference
Female	<0.001^*∗∗∗*^	2.322 (1.601–3.367)	<0.001^*∗∗∗*^	1.989 (1.349–2.935)
Major				
Nonmedical		Reference		
Medical	0.701	0.933 (0.657–1.326)		
Education level				
Undergraduate	0.217	Reference		
Master's	0.086	1.425 (0.951–2.137)		
Doctoral	0.934	1.023 (0.591–1.770)		
Oral habits				
Without		Reference		Reference
With	<0.001^*∗∗∗*^	3.795 (2.586–5.567)	<0.001^*∗∗∗*^	3.482 (2.359–5.138)

^*∗∗∗*^ *P* < 0.001.

**Table 5:** Results of the multivariate linear regression model (*N* = 505) using the total score of the Oral Health Impact Profile for TMDs (OHIP-TMDs) as the dependent variables.

Variable	Correlation analysis		Multivariate linear regression				
	*R*	*P*	*B*	Std. error	Beta (standardized)	95% confidence interval for *B*	Adjusted *P*
(Constant)			4.516	1.879		0.825–8.208	0.017
Age	0.026	0.557					
Gender	0.103	0.021^*∗*^					
Male					Reference		
Female			0.451	1.118	0.018	(−1.745)–2.647	0.687
Major	0.069	0.119					
Nonmedical							
Medical							
Education level	0.072	0.104					
Undergraduate							
Master's							
Doctoral							
Oral habits	0.243	<0.001^*∗∗∗*^					
Without					Reference		
With			4.388	1.104	0.177	2.219–6.558	<0.001^*∗∗∗*^
*R* square							
Adjusted *R* square			0.033/0.029				

^*∗*^ *P* < 0.05 and ^*∗∗∗*^*P* < 0.001.

## Data Availability

The data used to support the findings of this study are available from the corresponding author upon reasonable request.

## Supplementary Materials

Supplementary Materials Table S1. The summed scores of the PHQ-4 and FAI scale and the psychological status, and the presence of TMD in non-medical (*N* = 241) and medical students (*N* = 264).

## References

[1] List T., Jensen R. H. (2017). Temporomandibular disorders: old ideas and new concepts. *Cephalalgia*.

[2] Wieckiewicz M., Grychowska N., Wojciechowski K. (2014). Prevalence and correlation between TMD based on RDC/TMD diagnoses, oral parafunctions and psychoemotional stress in polish university students. *BioMed Research International*.

[3] Al-Khotani A., Naimi-Akbar A., Albadawi E., Ernberg M., Hedenberg-Magnusson B., Christidis N. (2016). Prevalence of diagnosed temporomandibular disorders among Saudi Arabian children and adolescents. *The Journal of Headache and Pain*.

[4] Christidis N., Lindström Ndanshau E., Sandberg A., Tsilingaridis G. (2019). Prevalence and treatment strategies regarding temporomandibular disorders in children and adolescents-a systematic review. *Journal of Oral Rehabilitation*.

[5] Lövgren A., Österlund C., Ilgunas A., Lampa E., Hellström F. (2018). A high prevalence of TMD is related to somatic awareness and pain intensity among healthy dental students. *Acta Odontologica Scandinavica*.

[6] Santana G. L., de Melo Júnior P. C., Aroucha J. M. C. N. L. (2019). Prevalence of TMD and level of chronic pain in a group of Brazilian adolescents. *PLoS One*.

[7] Gauer R. L., Semidey M. J. (2015). Diagnosis and treatment of temporomandibular disorders. *American Family Physician*.

[8] Poveda Roda R., Bagan J. V., Diaz Fernandez J. M., Hernandez Bazan S., Jimenez Soriano Y. (2007). Review of temporomandibular joint pathology. Part I: classification, epidemiology and risk factors. *Med Oral Patol Oral Cir Bucal*.

[9] Kmeid E., Nacouzi M., Hallit S., Rohayem Z. (2020). Prevalence of temporomandibular joint disorder in the Lebanese population, and its association with depression, anxiety, and stress. *Head & Face Medicine*.

[10] Perrotta S., Bucci R., Simeon V., Martina S., Michelotti A., Valletta R. (2019). Prevalence of malocclusion, oral parafunctions and temporomandibular disorder-pain in Italian schoolchildren: an epidemiological study. *Journal of Oral Rehabilitation*.

[11] Bueno C. H., Pereira D. D., Pattussi M. P., Grossi P. K., Grossi M. L. (2018). Gender differences in temporomandibular disorders in adult populational studies: a systematic review and meta-analysis. *Journal of Oral Rehabilitation*.

[12] Pedrelli P., Nyer M., Yeung A., Zulauf C., Wilens T. (2015). College students: mental health problems and treatment considerations. *Academic Psychiatry*.

[13] Minghelli B., Morgado M., Caro T. (2014). Association of temporomandibular disorder symptoms with anxiety and depression in Portuguese college students. *Journal of Oral Science*.

[14] Zhang W. R., Wang K., Yin L. (2020). Mental health and psychosocial problems of medical health workers during the COVID-19 epidemic in China. *Psychotherapy and Psychosomatics*.

[15] Lowe B., Wahl I., Rose M. (2010). A 4-item measure of depression and anxiety: validation and standardization of the Patient Health Questionnaire-4 (PHQ-4) in the general population. *Journal of Affective Disorders*.

[16] Zacca E. R., Crespo M. I., Acland R. P. (2015). Aging impairs the ability of conventional dendritic cells to cross-prime CD8 + *T* cells upon stimulation with a TLR7 ligand. *PLoS One*.

[17] Nomura K., Vitti M., Oliveira A. S. d. (2007). Use of the Fonseca’s questionnaire to assess the prevalence and severity of temporomandibular disorders in Brazilian dental undergraduates. *Brazilian Dental Journal*.

[18] Zhang M. J., Yap A. U., Lei J., Fu K. Y. (2020). Psychometric evaluation of the Chinese version of the fonseca anamnestic index for temporomandibular disorders. *Journal of Oral Rehabilitation*.

[19] Pires P. F., de Castro E. M., Pelai E. B., de Arruda A. B. C., Rodrigues-Bigaton D. (2018). Analysis of the accuracy and reliability of the short-form fonseca anamnestic index in the diagnosis of myogenous temporomandibular disorder in women. *Brazilian Journal of Physical Therapy*.

[20] Yap A. U., Qiu L. Y., Natu V. P., Wong M. C. (2020). Functional, physical and psychosocial impact of temporomandibular disorders in adolescents and young adults. *Medicina Oral, Patología Oral y Cirugía Bucal*.

[21] He S. L., Wang J. H. (2015). Validation of the Chinese version of the oral health impact profile for TMDs (OHIP- TMDs-C). *Medicina Oral, Patología Oral y Cirugía Bucal*.

[22] Ujin Yap A., Cao Y., Zhang M. J., Lei J., Fu K. Y. (2021). Age-related differences in diagnostic categories, psychological states and oral health-related quality of life of adult temporomandibular disorder patients. *Journal of Oral Rehabilitation*.

[23] Zhang M., Zhang J., Zhang F., Zhang L., Feng D. (2018). Prevalence of psychological distress and the effects of resilience and perceived social support among Chinese college students: does gender make a difference?. *Psychiatry Research*.

[24] Ivica A., Galić N. (2014). Attitude towards oral health at various colleges of the university of Zagreb: a pilot study. *Acta Stomatologica Croatica*.

[25] Yao K., Yao Y., Shen X., Lu C., Guo Q. (2019). Assessment of the oral health behavior, knowledge and status among dental and medical undergraduate students: a cross-sectional study. *BMC Oral Health*.

[26] Oliveira M. T. d., Bittencourt S. T., Marcon K., Destro S., Pereira J. R. (2015). Sleep bruxism and anxiety level in children. *Brazilian Oral Research*.

[27] Quek T. T., Tam W. W., Tran B. X. (2019). The global prevalence of anxiety among medical students: a meta-analysis. *International Journal of Environmental Research and Public Health*.

[28] Karibe H., Shimazu K., Okamoto A., Kawakami T., Kato Y., Warita-Naoi S. (2015). Prevalence and association of self-reported anxiety, pain, and oral parafunctional habits with temporomandibular disorders in Japanese children and adolescents: a cross-sectional survey. *BMC Oral Health*.

[29] Dawson A., Ghafouri B., Gerdle B., List T., Svensson P., Ernberg M. (2015). Effects of experimental tooth clenching on pain and intramuscular release of 5-HT and glutamate in patients with myofascial TMD. *The Clinical Journal of Pain*.

[30] Natu V. P., Yap A. U. J., Su M. H., Irfan Ali N. M., Ansari A. (2018). Temporomandibular disorder symptoms and their association with quality of life, emotional states and sleep quality in South-East Asian youths. *Journal of Oral Rehabilitation*.

