# Supplementary material for: College Students with Oral Habits Exhibit Worse Psychological Status and Temporomandibular-Related Quality of Life: A Correlational Study
Source: Pain Res Manag. 2022 May 11;2022:6079241. doi: 10.1155/2022/6079241 (PMC9117048; doi:10.1155/2022/6079241)
Supplement: Supplementary Materials — Table S1. The summed scores of the PHQ-4 and FAI scale and the psychological status, and the presence of TMD in non-medical (N = 241) and medical students (N = 264). [file 6079241.f1.docx]

**Table S1.** The summed scores of the PHQ-4 and FAI scale and the psychological status and the presence of TMD in non-medical (N=241) and medical students (N=264).

| **Variable** | **Non-medical**  **(N=241)** | **Medical**  **(N=264)** | ***P*** |
| --- | --- | --- | --- |
| Average score (point) |  |  |  |
| The PHQ-4 | 3.46 ± 2.88 | 2.66 ± 2.35 | 0.004^**^ |
| The FAI | 20.12 ± 18.96 | 18.26 ± 16.80 | 0.438 |
| Psychological distress |  |  | < 0.001^***^ |
| Without | 88 (36.51%) | 137 (51.89%) |  |
| With | 153 (63.49%) | 127 (48.11%) |  |
| TMD |  |  | 0.647 |
| No | 131 (54.36%) | 148 (56.06%) |  |
| Mild | 85 (35.27%) | 97 (36.74%) |  |
| Moderate | 19 (7.88%) | 15 (5.68%) |  |
| Severe | 6 (2.49%) | 4 (1.52%) |  |

^*^ *P* < 0.05; ^**^ *P* < 0.01; ^***^ *P* < 0.001
